# In vivo imaging of injured cortical axons reveals a rapid onset form of Wallerian degeneration

**DOI:** 10.1186/s12915-020-00869-2

**Published:** 2020-11-18

**Authors:** Alison Jane Canty, Johanna Sara Jackson, Lieven Huang, Antonio Trabalza, Cher Bass, Graham Little, Maria Tortora, Shabana Khan, Vincenzo De Paola

**Affiliations:** 1 Wicking Dementia Research and Education Centre, University of Tasmania, Hobart, Australia; 2 Dementia Research Institute at Imperial College, Department of Brain Sciences, Imperial College London, London, W12 0NN UK; 3 Institute of Clinical Sciences, Faculty of Medicine, Imperial College London, London, W12 0NN UK; 4 Medical Research Council London Institute of Medical Sciences, London, W12 0NN UK

**Keywords:** Wallerian degeneration, Brain, Cortex, Cortical axons, In vivo imaging, Synapses, Laser microsurgery, Axon fragmentation, Axon dieback, NAD^+^, Acute Axonal Degeneration

## Abstract

**Background:**

Despite the widespread occurrence of axon and synaptic loss in the injured and diseased nervous system, the cellular and molecular mechanisms of these key degenerative processes remain incompletely understood. Wallerian degeneration (WD) is a tightly regulated form of axon loss after injury, which has been intensively studied in large myelinated fibre tracts of the spinal cord, optic nerve and peripheral nervous system (PNS). Fewer studies, however, have focused on WD in the complex neuronal circuits of the mammalian brain, and these were mainly based on conventional endpoint histological methods. Post-mortem analysis, however, cannot capture the exact sequence of events nor can it evaluate the influence of elaborated arborisation and synaptic architecture on the degeneration process, due to the non-synchronous and variable nature of WD across individual axons.

**Results:**

To gain a comprehensive picture of the spatiotemporal dynamics and synaptic mechanisms of WD in the nervous system, we identify the factors that regulate WD within the mouse cerebral cortex. We combined single-axon-resolution multiphoton imaging with laser microsurgery through a cranial window and a fluorescent membrane reporter. Longitudinal imaging of > 150 individually injured excitatory cortical axons revealed a threshold length below which injured axons consistently underwent a rapid-onset form of WD (roWD). roWD started on average 20 times earlier and was executed 3 times slower than WD described in other regions of the nervous system. Cortical axon WD and roWD were dependent on synaptic density, but independent of axon complexity. Finally, pharmacological and genetic manipulations showed that a nicotinamide adenine dinucleotide (NAD^+^)-dependent pathway could delay cortical roWD independent of transcription in the damaged neurons, demonstrating further conservation of the molecular mechanisms controlling WD in different areas of the mammalian nervous system.

**Conclusions:**

Our data illustrate how in vivo time-lapse imaging can provide new insights into the spatiotemporal dynamics and synaptic mechanisms of axon loss and assess therapeutic interventions in the injured mammalian brain.

## Background

Axon degeneration leads to loss of connectivity and brain function upon trauma including head injury and stroke. Understanding the mechanisms of axon degeneration to either prevent or delay it, therefore, represents a promising avenue to develop effective therapies for these brain disorders [1].

Axonal injury is followed by degeneration of the distal part of the axon that is detached from the soma. This tightly controlled process of axon self-elimination following a lesion is termed WD [2] and has been intensively studied and characterised with the aim of devising new strategies to treat neurodegenerative and other diseases [1], which typically manifest with prominent local axon degeneration preceding cell body loss. Even though the triggering signals may be different, the execution phase of WD and axon degeneration in neurodegenerative diseases is conserved [3–7]. This common final axon removal step includes degradation of axoplasmic neurofilaments and microtubules, axon thinning, beading, fragmentation and clearance by resident glial cells [2, 8, 9].

Due to the limitations of current techniques, several aspects of WD remain to be fully established. WD is characterised by a protracted and variable lag phase before fragmentation which can take 24–48 h [3, 10–13]. Previous studies have suggested that the progression of WD depends on the length of the disconnected segment, with longer segments taking longer to degenerate than shorter ones [7, 12]. This length-based mechanism is consistent with the presence of axon protective factors such as NAD^+^ or the NAD^+^ synthesising enzymes, which when depleted from the axon trigger the process of fragmentation [14–17], with longer disconnected segments having inherently higher levels of such protective factors compared to shorter ones. This idea has been difficult to test because of the challenge of measuring the length of axons before a lesion in the living brain or spinal cord.

Existing, widely used experimental models are focussed on injured large myelinated fibres of the optic nerve [18, 19], spinal cord [20–25] and PNS [13, 26–29]. In particular, in vivo optical imaging has enabled fundamental discoveries on axon degeneration in the optic nerve [18] and spinal cord [20, 23, 25], e.g. the first description of Acute Axonal Degeneration (AAD), a form of WD occurring within minutes of injury, and of clinical relevance, as it removes portions of axon still connected to the soma and synapses near the lesion site [20], or of axon branch specific degeneration [25] (reviewed in [30], [31]). Whether similar or different mechanisms underlie the degeneration process in complex axonal networks such as those of the adult brain, however, is unknown. In particular, we lack critical information on the potential regulatory roles of axon branching and synaptic density and on how they might influence the degeneration process.

Most investigations of axon degeneration use cytosolic or cytoskeletal reporters [13, 18, 20, 22, 23, 27–29, 32], and as such, the kinetics and mechanisms of fragmentation of the surrounding axolemma remain unexplored. There are a handful of reports—in cultured peripheral neurons and the drosophila nervous system—noting increased resilience of the axonal membrane compared to cytosolic and cytoskeletal axonal components (microtubules, neurofilaments), which are affected first [4, 15]. To ensure the progression of axon degeneration can be followed to completion, it is therefore necessary to characterise the dynamics of axolemmal degeneration in vivo.

Axons constitute the largest neuronal compartment, surpassing the size of the cell body 1000-fold, in both length and surface area [33]. They can extend for up to 10 cm in the mouse brain [34] and branch thousands of times to reach all their targets [35]. As a result, it has not been possible to follow the degeneration process for individual unmyelinated cortical axons, an intricate fibre network vulnerable in many brain disorders, leaving the detailed spatiotemporal progression and synaptic mechanisms uncharted. High-energy lasers can be non-invasively directed with high precision to specific axonal segments, causing localised axonal breakage [25]. Laser microsurgery and live imaging have been successfully combined in the past to study axon regeneration in *C. elegans* [36] and the mouse brain [37–39], and for the study of spinal cord [23, 40] and retinal ganglion cell nerve injury [41], as well as stroke [42].

Here, we established and validated the combination of in vivo multiphoton time-lapse microscopy with laser microsurgery and a fluorescent membrane reporter to capture, for the first time, the cellular dynamics and regulatory mechanisms of cortical axon degeneration for up to 6 days post injury. We report four main findings: First, we identified a threshold length (~ 600 μm) below which damaged axonal endings consistently started fragmenting on average 20 times earlier than longer segments. Segments longer than ~ 600 μm degenerated with kinetics more in line with WD described in other regions of the nervous system. Second, cortical axon WD depended on synaptic density but not on the complexity of the axonal arbour. Third, AAD was less prominent in cortical unmyelinated axons than in spinal cord axons. Finally, a local NAD^**+**^-dependent pathway delayed cortical WD, independently of gene transcription in the damaged neuron. Our data support the use of longitudinal multiphoton in vivo imaging of fluorescent membrane markers and laser microsurgery to gain new insights into axon and synaptic degeneration and evaluate treatment strategies in the injured cerebral cortex.

## Results

### Single-axon-resolution imaging of Wallerian degeneration in the living brain

**Fig. 1:**
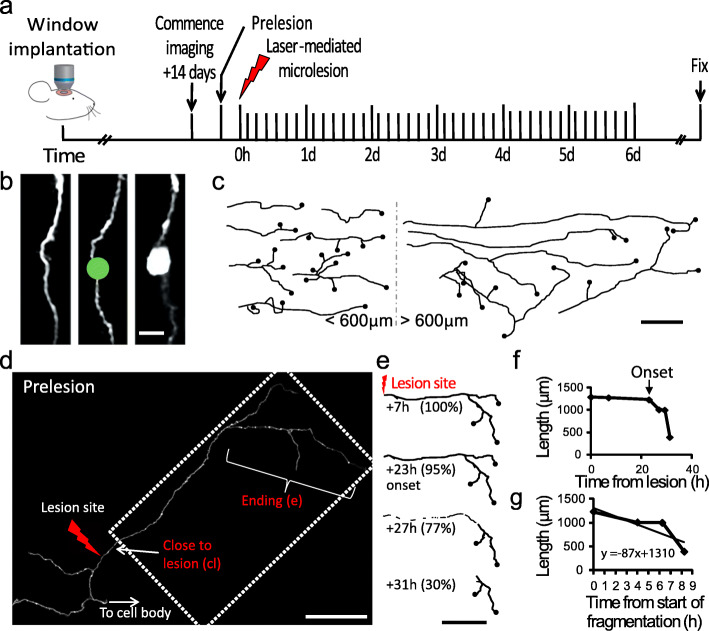
Single-axon-resolution imaging of axon degeneration in the living brain. **a** Timeline of imaging and injury to study cortical axon degeneration in vivo. **b** A region of axon ready for lesioning (left); a binary mask (4 μm) is placed over the axon (green circle) and a focused laser beam used to induce axotomy, verified by imaging immediately after lesion (right). Proximal is up and distal is down. **c** Representative drawings of axon segments before lesioning used throughout the study. Black filled circles indicate terminal axon endings. **d** A representative axon before lesioning, total length 1289 μm. **e** Representative drawings of the disconnected portion of the axon in the dotted box in **d** illustrating axonal degeneration over selected time points. Time in hours post-lesion and percentage of axon remaining are indicated. **f** Representative example of quantification of the degeneration onset (23 h) and **g** calculation of fragmentation rate for the axon in **d**, **e** (1.45 μm/min). Scale bar = 5 μm (**b)**, 150 μm (**c**), 200 μm (**d**) and 300 μm (**e**)

In order to study the progression of WD at single axon resolution over several days in the mammalian brain, we implanted cranial windows over the mouse somatosensory cortex [43] and combined 2-photon imaging with a well-established microlesion paradigm based on femtosecond laser-mediated subcellular ablation [36, 40] (Fig. 1a, b). To monitor the integrity of the axonal membrane in real-time [4, 7], we opted for a membrane-targeted form of GFP under the Thy1.2 promoter, which in the cerebral cortex drives expression of the transgene predominantly in excitatory cells of cortical layers 2/3/5/6 and thalamic origin [44]. To demonstrate the power of this methodology for the study of cortical axon WD, we describe several applications aimed at shedding light on the time course and regulatory factors of degeneration in injured neocortical circuits.

### A rapid onset form of Wallerian degeneration

**Fig. 2.**
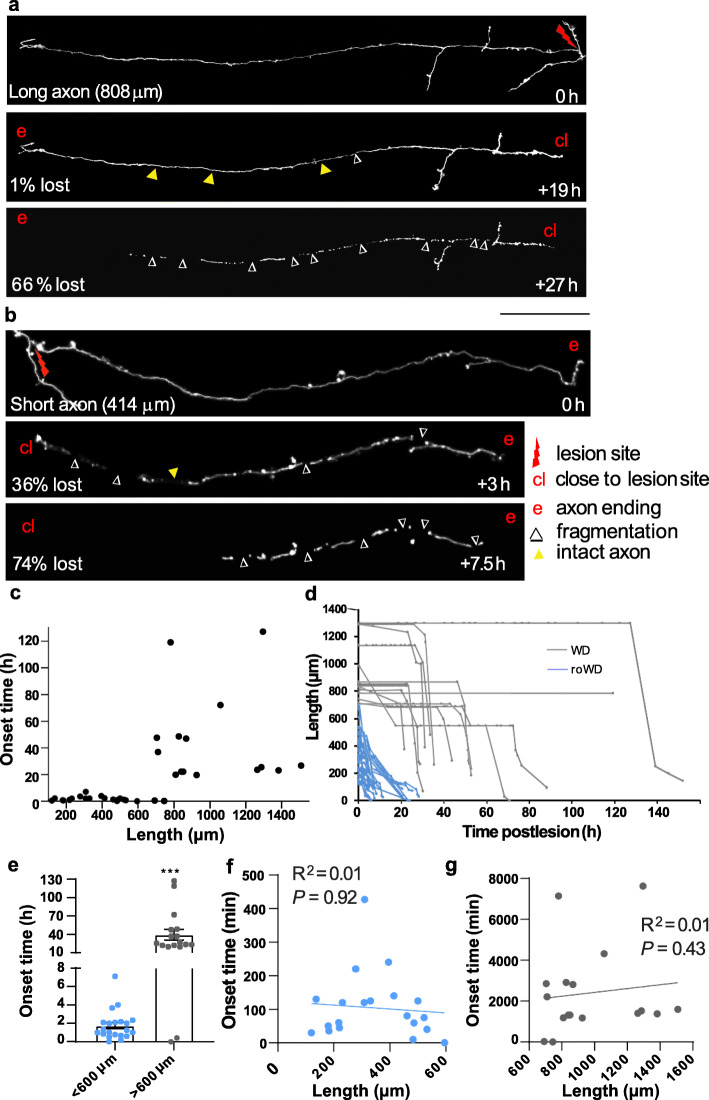
A rapid onset form of Wallerian degeneration in the adult cortex. **a** Representative images of a long axon ending undergoing Wallerian degeneration (WD) with time post-lesion and percentage of axon lost indicated. **b** Representative images of a shorter axon ending undergoing rapid onset WD (roWD) with time post-lesion and percentage of axon lost indicated. **c** Scatter plot indicating the presence of two axonal populations with different onset time of fragmentation. **d** Surviving length of each in vivo injured axon (*n* = 38) over the time points indicated illustrating a population undergoing roWD (blue lines). **e** Histogram demonstrating significant early onset time of fragmentation of axons < 600 μm in length (roWD, blue) compared to longer axons (WD, grey). Mean ± SEM. Mann Whitney *U* test. ****p < 0.0001. **f**, **g** No correlation between roWD onset time and length (f, *n* = 21 axons < 600 μm from 13 mice, *p* > 0.05) or for longer axons undergoing WD (g, *n* = 17 axons > 600 μm from 14 mice, *p* > 0.05). Scale bar: 135 μm (**a**), 70 μm (**b**). For further examples of fragmenting axons as in panels **a**-**b**, see Additional file 1

Individual cortical axons were identified as terminal endings and transected using a laser-mediated lesion (Fig. 1b–d) [37–39] to generate disconnected axon segments of known length from the edge of the lesion site to the terminal ending (range 0.12–1.5 mm). The onset (Fig. 1e, f) and rate (Fig. 1e, g) of fragmentation were calculated for each axon by detailed analysis of sequentially captured images. To determine the onset and rate of fragmentation, severed axons were imaged at regular intervals of up to 5 h (or for a subset of 8 axons less than 2 h, see methods) and the time point of the imaging session immediately preceding the first sign of clearly visible break in the axon (i.e. fragmentation) recorded as the ‘onset time’. A decrease in length of more than 10 μm over two consecutive sessions and a clear break in the axon shaft was considered as degeneration. At each subsequent imaging session, the sum of the individual fragment lengths was expressed as a percentage of the length of the disconnected axon immediately prior to fragmentation. The gradient (m) of the resulting slope was used to calculate the fragmentation rate using the formula *y* = *mx* + *c* (where *c* is the *y* intercept). We found that shorter disconnected axons, < ~ 600 μm in length, started the degeneration process by fragmenting significantly earlier than longer segments, > ~ 600 μm in length (Fig. 2a, b, Additional file 1). Relating the length and onset times for 38 transected cortical axons suggested the presence of two types of degeneration responses (Fig. 2c).

Following this observation of bimodality within the axon length and fragmentation onset time data, we sought to rigorously test the existence of two populations. The two Gaussian components of these bimodal distributions were fitted using the R mixtools packages (Version 1.0.2). The mean and standard deviations were calculated for these modelled Gaussian components, and the upper 95% confidence intervals of the components with the lower mean within both the onset time and length measurements identified. This allowed for the classification of a subset of data points as significant outliers to the lower components of both onset time and length. This subclass of data points was found to have significantly higher mean onset times compared to the rest of the data (*p* < 0.01, Wilcoxon rank sum test). Indeed, tracking individual injured axons continuously for up to 153 h at < 5 h intervals to capture the onset of fragmentation showed that longer transected axonal segments (typically > 600  μm from the ending; mean = 973 ± 65 μm, *n* = 17 axons from 14 mice) consistently initiated fragmentation after 19–127 h in line with the relatively slow onset described in the WD literature (15 out of 17 examples, i.e. 88%, Fig. 2a, d, e). In contrast, shorter transected axonal segments (typically < 600 μm from the ending; mean = 339  ± 32 μm, *n* = 21 axons from 13 mice) consistently initiated fragmentation on average ~ 20 times earlier than the longer portions (20 out of 21 examples, i.e. 95%; Fig. 2b, d, e, < 600 μm, onset time = 1.7 ± 0.4 h, *n* = 21; > 600 μm, onset time = 40.0 ± 8.7 h, *n* = 17, *p* < 0.0001), via a process we termed rapid onset WD (roWD). However, despite falling into two clear groups, we found no correlation between the time of onset of fragmentation and the actual length of each disconnected axon ending *within each group* (Fig. 2f, < 600 μm undergoing roWD onset time versus length, *R*^2^ = 0.08, *p* > 0.05; Fig. 2g, > 600 μm undergoing WD, *R*^2^ = 0.07, *p* > 0.05).

**Fig. 3:**
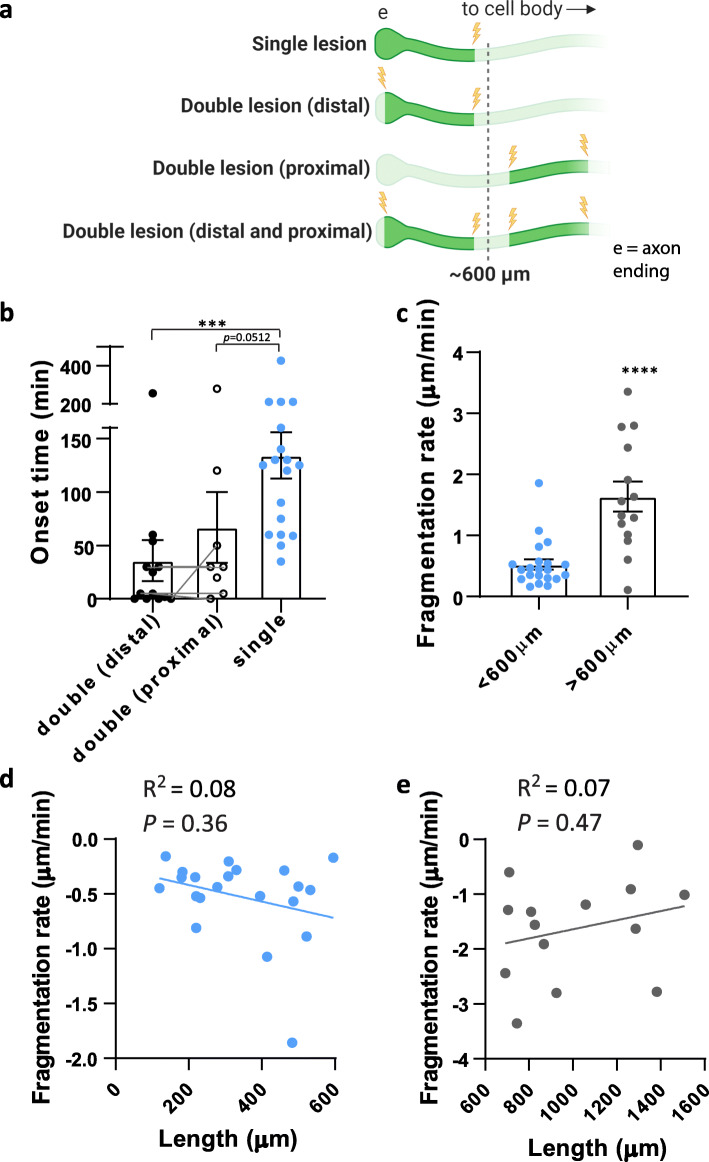
Probing axon degeneration kinetics in vivo. **a** Schematic illustrating single and double lesion experiments from different regions of the axon. **b** Average fragmentation onset time for double lesioned distal axons (black circles, *n* = 13 axons, 10 mice), double lesioned proximal axons (open circles, *n* = 8 axons, 7 mice) and single lesioned axons (blue circles, *n* = 18 axons, 9 mice). Grey lines link data points from double lesioned distal and proximal segments along the same axon (*n* = 5 axons, 3 mice). Wilcoxon test, *** *p* < 0.001. **c** Slower fragmentation rate of shorter axons undergoing roWD (blue points) compared to longer axons undergoing WD (grey points) after a single axonal lesion, **** *p* < 0.0001. **d** No correlation between roWD fragmentation rate and axon length (blue points, *n* = 21 axons, 13 mice, *p* > 0.05) or for longer axon WD fragmentation rate and axon length (**e**, grey points, *n* = 14 axons, 14 mice, *p* > 0.05)

These results are consistent with the possibility that the distal portion of the axon, within 600 μm from the ending, has a different competency for degeneration compared to more proximal locations (> 600 μm from the ending). To test this idea, we used laser microsurgery to cut equal-length segments from different regions along the axon—either located within 600 μm of the axon ending (distal), or further back along the axon shaft where the axon fragment generated was located more than 600 μm from the end (proximal). We induced lesions at either end of both segments so that both the distal and the proximal segments were comparable (double lesion (distal) and double lesion (proximal), Fig. 3a). For the double lesion (proximal) segment experiment, this also generated a second segment that contained the axon ending and was at least 600 μm in length—this segment was ignored for the purpose of this experiment. We found no difference in the degeneration onset time between these two groups of axon segments (double lesion (distal) and double lesion (proximal), *p* > 0.05, Fig. 3b). In some cases, we were able to cut two equal length axon segments separated by at least 600 μm from each other along the *same* axon (Fig. 3b; *n* = 5 axons from 3 mice, grey lines linking data points) and found again no difference in the degeneration onset (*p* > 0.05). These data suggest that the location of the disconnected axon segments along the axon does not affect the kinetics of the degeneration process.

An alternative possibility to explain the faster degeneration onset in shorter axon segments is that a threshold-dependent mechanism controls the kinetics of cortical roWD and WD [7]. This threshold model, whereby the initiation of axon degeneration could be mediated by the loss of a protective factor and/or the accumulation of a degeneration trigger (see discussion, and illustrated in an additional file (Additional file 2)), predicts that axonal segments of the same length with two cut ends would have earlier fragmentation onset than equal length segments having only one cut end, owing to two depletion/accumulation routes, instead of one.

So, we compared the double lesion (distal) data set (mean length = 187 ± 18 μm, *n* = 13 axons, 10 mice) with the original data collected for axon segments generated by a single lesion within a known distance of the terminal ending (Fig. 3a. single lesion, mean length = 310 ± 30 μm, *n* = 18 axons, 8 mice). The double lesion segments showed a significantly earlier onset of fragmentation compared to the single lesion axons (Fig. 3b, single lesion onset = 134 ± 24 min; double distal lesion onset = 34 ± 19 min; *p* = 0.001), as predicted by the threshold-dependent axon degeneration model. To further explore whether the double lesion had to flank the distal ending to generate this effect, we also compared the single lesion onset time to the double lesion (proximal) segments located more than 600 μm from the ending (*n* = 8 axons, 7 mice). Similarly, this showed a trend towards a significantly earlier onset time in the double (proximal) axons segments compared to the single lesions (Fig. 3b, single lesion onset = 134 ± 24 min; double proximal lesion onset = 66 ± 33 min; *p* = 0.0512). This supports a threshold-dependent model to explain the onset of degeneration in transected cortical axons.

To further investigate the degeneration kinetics of roWD and WD, using a single lesion paradigm, we compared the fragmentation rates of the surviving axon segments once fragmentation onset was detected. Surprisingly, the rate of fragmentation for shorter transected axonal segments (< 600 μm from the ending) was significantly slower than for longer transected axonal segments (Fig. 3c, < 600 μm, 0.5 ± 0.1 μm/min, *n* = 21 axons, 13 mice; > 600 μm, 1.6 ± 0.2 μm/min, *n* = 14 axons, 14 mice; *p* < 0.0001). Similar to the onset time, within each group, there was no correlation between the length of the disconnected axon segments and the fragmentation rate (Fig. 3d, e, < 600 μm, *R*^2^ = 0.08, *p* > 0.05; > 600 μm, *R*^2^ = 0.07, *p* > 0.05), again ruling out a linear length-based mechanism to explain the degeneration kinetics.

Using a similar image analysis approach, we investigated the site of the first observed fragmentation along injured cortical axons. Out of 32 axons, 10 started fragmenting close to the lesion site, 11 from the terminal ending furthest from the lesion, 5 along the entire length at the same time, 3 from the middle and 3 from both ends simultaneously, with different regions displaying comparable onset (close to lesion site, onset time = 7.3 ± 2.4 h, *n* = 26; distal end, onset time = 10.4 ± 2.9 h, *n* = 26, *p* > 0.05) and fragmentation speed (close to lesion end, 0.19 ± 0.035 μm/min, *n* = 16; distal end, 0.19 ± 0.038 μm/min, *n* = 16, *p* > 0.05). Intriguingly, when we divided the axons into two groups, shorter or longer than 600 μm, despite the low numbers, fragmentation in shorter axons undergoing roWD was significantly more likely to start at the distal axon ending (40% of lesioned axons) compared to longer axons undergoing WD (10% of lesioned axons; Chi squared test, *p* < 0.001).

### Cortical WD depends on bouton density but not axonal arbour complexity

**Fig. 4:**
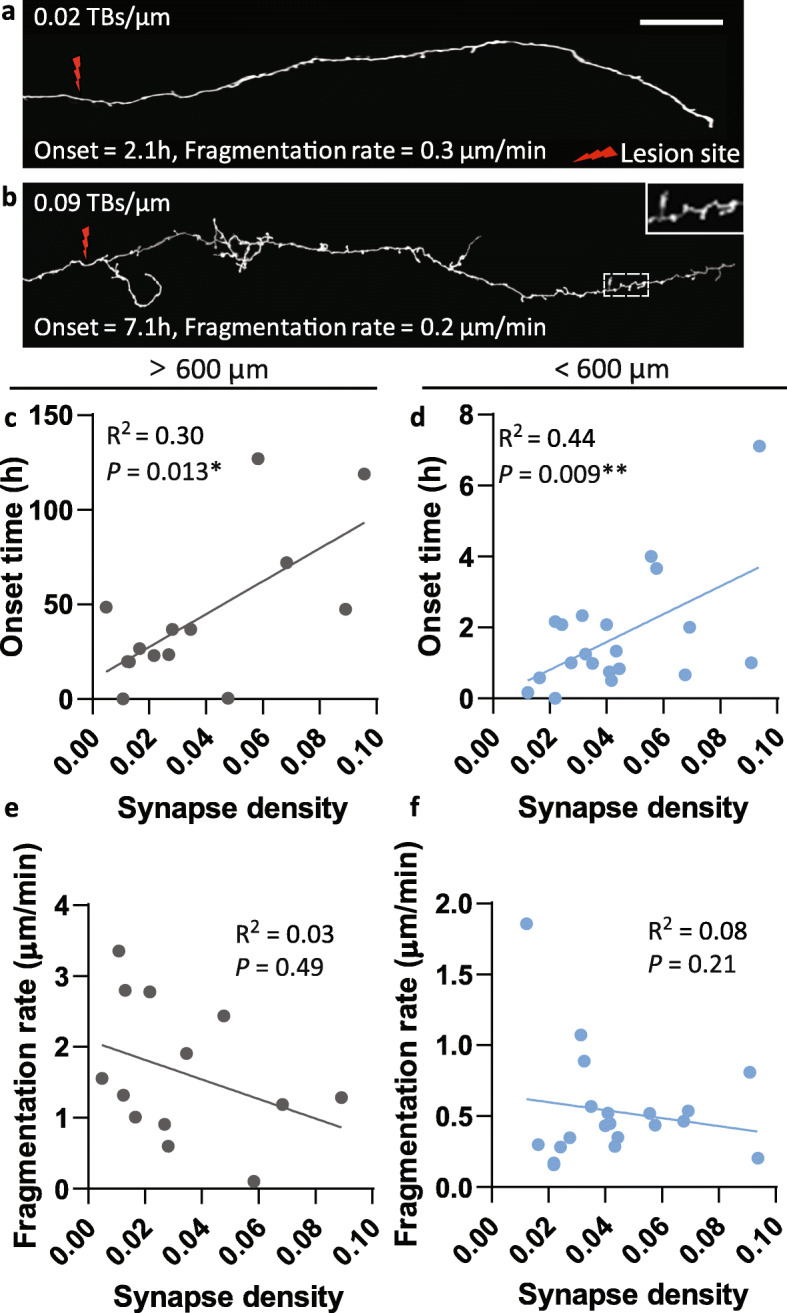
Synaptic boutons delay the onset of degeneration in cortical axons. **a**, **b** Representative images of axons with relatively low (**a**) and high (**b**) densities of *terminaux* boutons (TBs). **c**, **d** There is a significant correlation between bouton density and the onset of fragmentation for segments undergoing WD (**c**, *p* < 0.05, *n* = 13 axons, 13 mice) and for shorter axons undergoing roWD (**d**, *p <* 0.01, *n* = 20 axons, 12 mice). **e**, **f** There is no correlation between bouton density and fragmentation rate for segments undergoing WD (**e**, *p* > 0.05) or roWD (**f**, *p* > 0.05). Scale bar 40 μm (**a**, **b**)

Laser microsurgery and in vivo imaging of individual grey matter axons opens the way to study the role of axonal arborisation and synaptic architecture in WD, which could not be assessed in previous work on large myelinated fibres of the spinal cord, optic nerve or the PNS. So, we asked how the density of boutons along cortical axons and the complexity of axonal arborisation affects roWD and WD. We hypothesised that axons with a relatively high density of synaptic contacts may take longer to degenerate than axons with lower synaptic density due to the molecular complexity within the axon and the additional time required to disassemble the synaptic machinery [15], even though axons with a greater synapse density would have a greater energetic burden. The bouton density (range 0.0048–0.0957 boutons/μm) of 33 axons was quantified on the basis of morphology (*terminaux* boutons TB) in the distal axon segment prior to the laser micro-lesion, with all protrusions between 1 and 5 μm extending from the midline of the axonal shaft considered as boutons. These axonal structures have previously been confirmed as forming complete synapses [38, 44, 45]. The bouton density was correlated with the onset and rate of fragmentation of the same segments (Fig. 4a, b). For segments > 600 μm undergoing WD, bouton density significantly correlated with the onset (*R*^2^ = 0.30, *p* < 0.05; Fig. 4c) but not the rate of fragmentation (*R*^2^ = 0.03, *p* > 0.05; Fig. 4e, *n* = 13 axons, 13 mice). For segments < 600 μm undergoing roWD, bouton density also significantly correlated with the onset (*R*^2^ = 0.44 *p* < 0.01; Fig. 4d), but not with the rate of fragmentation (*R*^2^ = 0.08, *p* > 0.05; Fig. 4f, *n* = 20 axons, 12 mice). These data support the hypothesis that the onset of fragmentation in axons undergoing degeneration is dependent on the synaptic connectivity within the axon segment, where higher bouton density results in a later onset of fragmentation but has no significant relationship to the kinetics of fragmentation once commenced.

Investigation of kinetics in branched and unbranched axons showed no difference in onset times (unbranched > 600 μm, 24.7 ± 8.0 h, *n* = 5 axons, 5 mice; branched > 600 μm, 24.3 ± 7.7 h, *n* = 5 axons, 5 mice, *p* > 0.05; unbranched < 600 μm, 1.80 ± 0.45 h, *n* = 15 axons, 8 mice; branched < 600 μm, 1.81 ± 0.74 h, *n* = 4 axons, 3 mice, *p* > 0.05) or fragmentation rates (unbranched > 600 μm, 1.81 ± 0.39 min; branched > 600 μm, 1.92 ± 0.49 min, *p* > 0.05; unbranched < 600 μm, 0.44 ± 0.06 min; branched < 600 μm, 0.54 ± 0.12 min, *p* > 0.05), suggesting that axonal arbor complexity does not play a major role in cortical axon degeneration kinetics.

### AAD is less prominent than roWD and WD in injured cortical axons in vivo

**Fig. 5:**
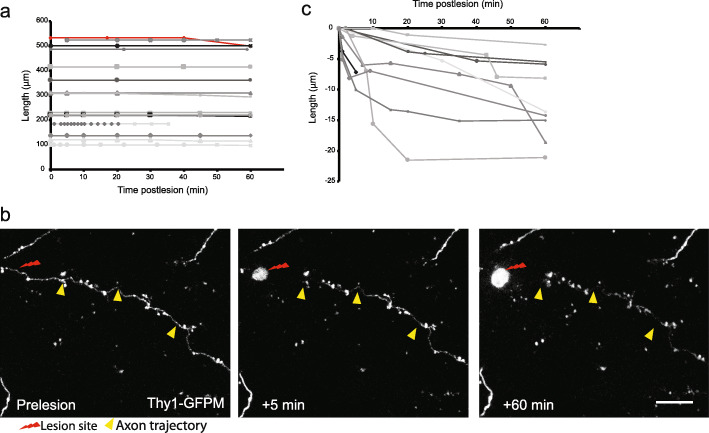
AAD is negligible in injured cortical axons in vivo. **a** No evidence of AAD within 60 min of injury when the length of severed cortical axon distal segments is monitored with a membrane marker (Thy1-L15). Note length stability in all but one axon (red line), indicating absence of fragmentation over the first 60 min post-lesion (*n* = 16 axons, 7 mice). **b** Example of the retraction of the severed axon away from the lesion site when observed with a cytosolic GFP marker (GFP-M line). Note the absence of fragmentation over the first 60 min post-lesion. Arrowheads indicate intact axon. Axon ending is on the right edge of the frame. Scale bar = 10 μm. **c** Quantification of the distance from the lesion site to the distal axon stump in cytosolic GFP labelled axons (Thy1-GFPM line) at the indicated post-lesion times (*n* = 10 axons, 7 mice)


Frequent regular imaging immediately after transection revealed little evidence of cortical axon fragmentation occurring within the first 60 min post-lesion, either proximal [39] or distal to the lesion site as illustrated in an additional movie file (see Additional file 3), with all but one disconnected axonal segments (37 out of 38, 97%) exhibiting a delay in fragmentation onset of at least 1 h (Fig. 5a). Significant axonal fragmentation (~ 200–300 μm) on both sides of the lesion within few minutes of injury, however, was reported in the myelinated fibres of spinal cord [20] and optic nerve [18]. This difference could be due to different lesion methods (laser versus mechanical axotomy), markers used (axonal membrane versus cytosolic) or intrinsic characteristics of the different neuronal populations in the nervous system (cerebral cortex, spinal cord, optic nerve).

**Additional file 3.** Canty et al. Additional file 3.mov. Time-lapse movie of axon degeneration in the living mouse brain. This movie shows the degeneration of a distal axon fragment over time in the mouse cerebral cortex as viewed via a cranial window preparation. Post-lesion time stamps are indicated on each frame.

To investigate these possibilities, we performed laser axotomies and in vivo imaging in both the ascending dorsal white matter tracts in the spinal cord (where AAD was first described [20]) and in cortical axons using either cytosolic (GFP-M line [46]) or axolemmal GFP markers (L15 line [47, 48]), expressed under the Thy 1.2 promoter. In vivo time-lapse imaging of individual ascending spinal axons (see Additional file 4) showed that while the axoplasm (cytosol and cytoskeletal components) fragmented within minutes in the proximity of the spinal cord lesion (GFP-M line, axoplasm degeneration onset = 29.6 ± 4 min; range 16–56 min, *n* = 9 axons, 5 mice) (Additional file 4a, c) consistent with previously described AAD [23], the membrane near the lesion site remained intact for up to ~ 4 h (L15 line, axolemmal onset = 139.9 ± 22 min; range 58–228 min, *n* = 9 axons, 5 mice; *p* < 0.001 compared to axoplasmic onset) (Additional file 4b, c). Analysis of the position of the two severed axon tips, proximal and distal to the lesion site, showed that the axonal membrane underwent almost no retraction from the lesion site in the first 60 min on either side of the lesion (L15 line, proximal tip 8.2 ± 1.7 μm; range 4.1–11.3 μm; distal tip 8.9 ± 3.0 μm, range 2.7–15.7 μm, *n* = 5 axons), compared to the cytosolic labelled axons which underwent significant retraction on both sides of the lesion site between 30 and 60 min post lesion (GFP-M line, proximal tip 85.0 ± 37.9 μm; range 13.5–264.2 μm; distal tip 82.4 ± 25.9 μm, range 22.1–185.4 μm*, n* = 7 axons) (Additional file 4d).

When we returned to investigate the dynamics of cytosolic GFP in cortical axons using the GFP-M line, the axoplasm of the distal disconnected cortical axon segments appeared to slowly retract away from the lesion site over the first 60 min postlesion (Fig. 5b, c**;** 11.1 ± 1.9 μm; range 3–21 μm*; n* = 10 axons) [23, 24]. This is potentially due to leakage of GFP from the cut end immediately after the lesion and occurs before any frank fragmentation of the distal axon.

Interestingly, in cortical axons the fragmentation rate of membrane-bound GFP was much slower than the rate of cytosolic GFP fragmentation (0.5 ± 0.1 μm/min versus 3.4 ± 0.7 μm/min, respectively; *p* < 0.05), and both were slower than rates measured from previous studies in the spinal cord [20] and phrenic nerve [12], which could potentially be explained by the longer sampling intervals used in our chronic in vivo imaging protocols.

Taken together, we describe novel approaches in the exploration of cortical axon WD in vivo. We identify a threshold axon length below which the lag phase before cortical axon fragmentation (here called ‘onset’) is one order of magnitude earlier, and the fragmentation rate significantly slower than for WD, in a process we call roWD. In addition, in contrast to AAD [18, 20], roWD removes the entire severed distal axon rather than just a portion of it adjacent to the lesion site and does not occur on the proximal axonal segment (i.e. the portion still connected to the cell body), revealing spatiotemporal differences in degeneration between roWD and WD/AAD, summarised in an Additional file (see Additional file 5).

### Cortical axon WD is controlled by a local NAD^+^-dependent pathway

**Fig. 6:**
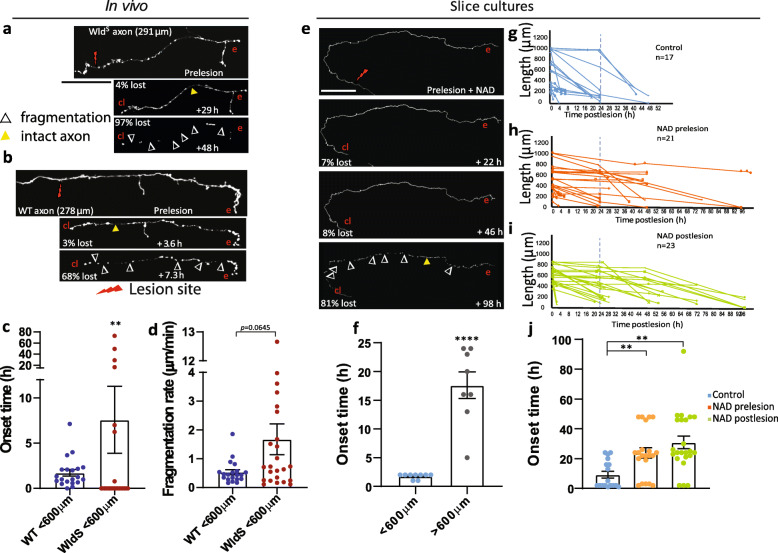
A local NAD^+^-dependent pathway controls cortical axon WD. **a**, **b** Representative examples of a lesioned Wld^S^–Thy1-L15 and WT axons of comparable distal length. **c** Wld^S^ axons have a significantly later fragmentation onset time than WT axons, Wld^S^, *n* = 24 axons, 11 mice; WT *n* = 21 axons, 13 mice, ** *p* < 0.01. **d** The fragmentation rates are not significantly different in WT and Wld^S^ axons although the data suggested a trend towards faster fragmentation in the Wld^S^ axons. **e** Representative images of a lesioned axon in cortical slice culture treated with NAD^+^. **f** Fragmentation onset time in lesioned axons in cortical slice cultures is significantly earlier in axons < 600 μm (roWD, blue, *n* = 9 axons, 5 slices, 5 mice) than axons > 600 μm in length (WD, grey, *n* = 8 axons, 6 slices, 2 mice), *** *p* < 0.001. **g**–**i** Degeneration of in vitro lesioned axons with no treatment (control, **g**), with addition of NAD^+^ prelesion (**h**) and postlesion (**i**). Blue dotted line shows the 24 h time point. **j** Axons are protected by NAD^+^ when added both before or after the lesion. Mean ± SEM. Mann Whitney *U* test. Scale bar 70 μm (**a**, **b**), 100 μm (**e**)

To explore the mechanism of roWD, we investigated NAD^**+**^-dependent signalling pathways, which have been implicated in both WD and AAD [20, 49, 50] and in modulating axon degeneration in several animal models of neurodegenerative disease [1]. To this end, we used the Wallerian degeneration slow (Wld^S^) mouse line overexpressing a stable version of the NAD^**+**^ synthesising enzyme NMNAT1 consisting of an additional 18 amino acid linkage to ubiquitination factor E4B, which has the effect of delaying axon degeneration [50, 51]. Mice with the Wld^S^ mutation have delayed onset of WD in both the peripheral and central nervous system [52, 53]. Wld^S^ mice were crossed with the Thy1-L15 line to produce heterozygous Wld^S^-Thy1-L15 progeny, and the cortical axon degeneration kinetics were determined using the aforementioned methodology in distal axons of < 600 μm in length in Wld^S^-Thy1-L15 (*n* = 24 axons, 11 mice, Fig. 6a) and compared with wild-type (WT) axons of comparable length (*n* = 21 axons, 13 mice, Fig. 6b). Wld^S^-Thy-L15 animals showed a significantly delayed onset of fragmentation (Fig. 6c, WT, 1.7 ± 0.4 h; Wld^S^-Thy1-L15, 7.6 ± 3.7 h, *p* < 0.01), indicating that roWD is regulated by NAD^+^-dependent pathways. There was no significant difference in fragmentation rate, although there was a trend for faster fragmentation in the Wld^S^-Thy1-L15 cortical axons (Fig. 6d; WT, 0.5 ± 0.1 μm/min; Wld^S^-Thy1-L15, 1.7 ± 0.5 μm/min, *p* = 0.0645). Interestingly, and unlike WT axons, once fragmentation commenced, it did so along the entire axonal length simultaneously.

To further probe NAD^+^-dependent regulation in cortical axon degeneration, we used cortical explant cultures to enable pharmacological manipulation of NAD^+^ levels [54]. Seventeen axons were lesioned, with mean length of 613 ± 80 μm, from 6 animals (range 0.12–5 mm) (Fig. 6e, f). We verified that the onset of fragmentation of lesioned cortical axons in slice culture was comparable with the onset in in vivo experiments with a significant delay in axons > 600 μm (Fig. 6f, g; onset = 1.8 ± 0.18 h, 9 axons < 600 μm; onset = 17.6 ± 2.48 h, 8 axons 600–1000 μm; *p* < 0.05). Fragmentation rates were not calculated for the in vitro experiments as the room temperature imaging conditions confounded the fragmentation kinetics [55]. Next, we tested whether the protective effect of NAD^+^ was due to a local action on the cut distal axon or to its potential role in modulating gene expression in the nucleus [21, 26, 49]. To distinguish between these possibilities, we first applied 1 mM NAD^+^ to a separate group of slice cultures 24 h before the lesions, which resulted in a robust delay in roWD onset (Fig. 6h, j; mean length 521 ± 51 μm, onset = 23.9 ± 3.5 h, *n* = 21 axons, 8 mice; *p* < 0.01 compared to untreated mean length 613 ± 80 μm, onset = 9.2 ± 2.2 h, *n* = 17 axons, 6 mice). To test the role of transcription-independent NAD^**+**^ effects in the damaged neurons [49], we also applied NAD^**+**^ immediately after the axon was cut. In this case, NAD^**+**^ was also able to delay the onset of degeneration (Fig. 6i, j; length = 613 ± 31 μm, onset = 30.9 ± 4.2 h, *n* = 23 axons, 10 mice; *p* < 0.01). These data suggest that the NAD^**+**^ action is local, in agreement with many other reports in the literature [50, 56, 57], rather than requiring gene expression in the axotomised neurons as shown for WD in a previous report in dissociated neuronal cultures [49].

## Discussion

Here, we studied the spatiotemporal patterns and mechanisms of WD in the cerebral cortex. We combined in vivo 2-photon time-lapse imaging of a fluorescent membrane tracer through a cranial window and laser-mediated lesions to track the full extent of WD for individual unmyelinated cortical axonal arbours (Fig. 1). We provide new insights into cortical axon degeneration: (1) segments below ~ 600 μm in length consistently undergo a faster form of WD (roWD); (2) synaptic density, but not arbor complexity, contributes to the timing of cortical axon WD; (3) AAD is negligible in lesioned cortical axons; and (4) similar to WD in other parts of the nervous system and AAD, an NAD^**+**^-dependent signalling pathway, independent of transcription, delays cortical axon roWD and WD.

In vivo optical imaging of spinal cord injury has provided unique insights into the mechanisms of axon degeneration and regeneration in recent years [20, 23, 58]. However, we are only now starting to exploit this tool to understand the principles of axon degeneration and regeneration in the mammalian brain [37–39, 59–61]. Limitations of optical methods through glass windows to induce local damage include the inability to model more complex types of injury conditions such as those found in traumatic brain injury or multiple sclerosis and the difficulty of getting therapeutic compounds into the brain in order to manipulate the local environment in vivo [62]. There are three main advantages of laser-mediated axonal transection compared to previously reported methods. Firstly, laser microsurgery reveals the entire degeneration process as close as 4–10 μm from the injury site along to the terminal ending of the axon [13, 38]. In contrast, following mechanical or needlestick injury, anatomical distortions mean that axonal changes can only be observed at a distance (> 100 μm) from the injury site. Secondly, local axonal injury can be induced without rupturing the dura mater and overlying blood vessels, decreasing contributions from inflammatory mediators, thereby reducing the complexity of cellular interactions and improving experimental reproducibility [38, 63]. Thirdly, several neurons, or even parts of a neuron [25], can be simultaneously targeted with minimal damage to the surrounding tissue [13, 36, 38, 64–67]. The combination of this lesion method with the selective monitoring of the axolemma, rather than the axoplasm as in previous studies, allowed us to accurately measure the timing of the execution phases of axon degeneration, ruling out instances of cytoplasmic fragmentation not directly accompanied by loss of membrane integrity (as reported previously [4, 15], and as our data suggest it is the case for AAD, Fig. 5). These results indicate that axoplasmic components such as microtubules and neurofilaments are the first to change in WD, while the axonal membrane persists for longer time, in agreement with these previous reports [4, 15]. We expect that this paradigm and its refinements, for example by simultaneously imaging cytoskeletal [68], mitochondria [69] and membrane markers, will be useful to investigate axonal degeneration in other mouse models of disease (e.g. Alzheimer’s disease) or human-mouse chimeric models, which employ patient-specific genetic backgrounds [31, 70].

To our knowledge, ours is the first study to tackle the challenge of monitoring the spatiotemporal dynamics, as well as shedding light on the mechanisms, of WD in the mammalian cerebral cortex at single axon resolution. We used a labelling strategy targeted mainly to excitatory unmyelinated axons originating in cortical layer 2/3/5/6 and the thalamus, because of their vulnerability in several neurodegenerative and acute injury conditions. The reported degeneration kinetics fall within published time frames for WD in the murine spinal cord [20], peripheral nerves [71] and optic nerve [18], but reveal that transected cortical axon segments < ~ 600 μm have a different spatial and temporal degeneration progression pattern compared to both WD and AAD. Even though only a small percentage (< 1%) of the axonal arbor when lesioned undergoes roWD, roWD affects up to 2–3 times more axon length than AAD [20] (see Additional file 5).

In earlier work in zebrafish larvae, the length of the disconnected axonal segment was not a critical parameter, not affecting the duration of the lag phase (which we here call onset) and only slightly influencing the fragmentation rate [13]. The difference in fragmentation rates between WD and roWD in mammalian cortical neurons (Fig. 2d, g) could reflect the natural variability of the degeneration process across species and the nervous system. It is well established that degeneration kinetics are variable between axons in adults [11], and even more so during development. For example, axotomised trigeminal axons in the developing zebrafish show embryonic stage-dependent differences in the time of the degeneration onset and a distinct lack of AAD [13]. Other axon subtypes, such as those of the zebrafish lateral line projections, do undergo a process resembling AAD in that a gap rapidly forms on both sides of the lesion, although no evidence of fragmentation was detected [29].

For cortical axons, the timing of both the initiation (onset, Fig. 2d, e) and execution phase (fragmentation kinetics, Figs. 2d and 3c) differs depending on a cut-off length of approximately 600 μm. Our data are not consistent with a linear length-based mechanism to explain the onset of cortical axon degeneration as suggested by others who find that longer disconnected segments take longer to commence degeneration, for example in transected zebrafish trigeminal axons [13]. A ‘threshold-dependent’ mechanism likely controls cortical roWD and WD as illustrated in an additional file (see Additional file 2). It could be either a ‘protective factor’ or a ‘degeneration trigger factor’ level-based threshold. For a ‘protective factor’ level-based threshold, the amount of a degeneration inhibitor (e.g. NAD^+^, or neuro-protective NAD synthesising enzyme Nmnat2 [14, 72, 73]) would decrease [74] below a critical threshold level before termination of the broken membrane resealing process (which can occur within 30 min post injury in vitro [75]), triggering rapid onset of fragmentation. In longer segments, the membrane from the cut end would reseal before depletion, and as such, these fragments persist. For a ‘degeneration trigger factor’ level-based threshold, a critical amount of a trigger such as calcium influx (e.g. via downstream effectors such as calpains [76]), mediated by extracellular calcium influx through the cut end [77, 78], would be rapidly reached in shorter segments which would therefore fragment quickly compared to longer segments. The rapid removal of short, damaged axonal segments from the neuropil may have evolved as a mechanism to rapidly restore brain homeostasis and keep the neuropil free from debris.

Our results on the regulation of cortical roWD and WD by NAD^+^-dependent mechanisms (Fig. 6) are consistent with previous data of delayed dopaminergic and striatal synaptic degeneration in Wld^S^ mice [79, 80], providing additional in vivo evidence that Wld^S^-based neuroprotective therapeutics may be developed not only for myelinated but also for unmyelinated, grey matter fibre degeneration. These results demonstrate further conservation of the molecular mechanisms controlling WD, despite different spatiotemporal patterns of degeneration in different areas of the mammalian nervous system [26].

Cortical axons have more delayed degeneration onset the higher the density of synaptic connections (Fig. 4). Synapse loss in neurodegeneration can occur independently of axonal and cell body loss, suggesting that different mechanisms regulate the degeneration of these compartments [81–83]. We hypothesise that the presence of synapses limits the depletion of axon protective factors or accumulation of degeneration triggers, possibly through either slower diffusion along the axon, or due to being crosslinked with synaptic machinery, prolonging the lag phase before fragmentation.

In the future, the in vivo optical imaging approach used here could lead to a better understanding of the molecular and cellular mechanisms that regulate cortical axon degeneration, a necessary step in the development of effective therapies to counteract or stabilise the progression of axon and synaptic loss in both the diseased and injured brain.

## Conclusions

We identify a rapid onset form of Wallerian Degeneration (roWD) in injured cortical axons shorter than 600 μm in length, which is dependent on synaptic density but not axon complexity nor the position of the severed segment along the axon shaft. In addition, we find that NAD^+^ signalling mechanisms regulate both cortical WD and roWD, demonstrating further conservation of the molecular mechanisms controlling WD in different areas of the mammalian nervous system. Our data highlight the potential for in vivo 2-photon time-lapse imaging to further investigate axonal degeneration and importantly to assess therapeutic interventions in the injured mammalian brain.

## Methods

### Animals

Adult male mice from Thy1-L15 [48] (membrane-targeted GFP, *n* = 32, 12–14 weeks), Thy1-GFPM [48] (cytosolic-targeted GFP, *n* = 12, 12–14 weeks) and the Wld^S^ line, which spontaneously expresses the slow Wallerian degeneration mutation (Wld^S^-Thy1-L15, *n* = 11, 12–14 weeks), were used for all in vivo imaging experiments unless otherwise specified. All mice were given access to food and water ad libitum and maintained in a 12-h light-dark cycle. All animal procedures were conducted at the Institute of Clinical Sciences, Imperial College London, by researchers holding a UK personal licence; conducted in accordance with the UK Animals (Scientific Procedures) Act 1986; and approved by the local Animal Welfare and Ethical Review Body (AWERB).

### Craniotomy

Cranial windows were surgically implanted over the somatosensory cortex as previously described [43]. Mice were anaesthetized with ketamine-xylazine intraperitoneal injection (0.083 mg/g ketamine, 0.0078 mg/g xylazine) and then administered intramuscular dexamethasone (0.02 ml at 4 mg/ml) to reduce inflammation, and subcutaneous bupivacaine (1 mg/kg), a local anaesthetic. A few drops of lidocaine (1% solution) were applied on the skull surface prior to a 5-mm diameter craniotomy being drilled over the somatosensory cortex. A glass coverslip was then placed over the craniotomy and sealed with glue and dental cement. The skull was subsequently covered in dental cement and a metal bar placed on top for positioning at the two-photon microscope. Mice were allowed to recover for 14 days prior to imaging.

### Two-photon imaging in vivo

A purpose built two-photon microscope (Prairie Technologies) equipped with a tunable Ti:Sapphire laser (Coherent) and PrairieView acquisition software was used for all imaging experiments. Mice were anaesthetized with isofluorane (1–1.5%) and secured to the microscope with the head metal bar attached to a custom-built fixed support. Lacri-lube was applied to the eyes to prevent dehydration and temperature maintained by a heating blanket. An Olympus 4X objective with a 0.13 numerical aperture (NA) was used to identify characteristic blood vessels to reliably locate regions of interest (ROIs) at each imaging time point. An Olympus 40X (NA = 0.8) water immersion objective was used to acquire several ROI stacks per animal (typically 100 × 100 μm field of view, 512 × 512 pixels, 1-μm step size). A pulsed 910-nm laser beam was used with typical power never exceeding 70 mW on the back focal plane. Each imaging session lasted a maximum of 90 min, after which animals recovered for at least 30 min before the next session began.

### Selection of cortical axons and laser-mediated axonal lesions in vivo

A total of 154 axons (93 in vivo and 61 in vitro) were lesioned and followed for up to 153 h in vivo and 96 h in vitro to assess axon degeneration kinetics. Axons located in the upper layers of the cortex were selected starting from their tips/endings, then followed along the axon shaft towards the cell body for as long as axon identification could be guaranteed, until the axon disappeared under a blood vessel, under the bone edges or could not be distinguished from neighbouring axons, which defined axonal segments of variable length. A laser micro-lesion protocol was optimised to induce a reproducible lesion as in [38, 39]. Briefly, the imaging laser was tuned to 800 nm and engineered to deliver a single spot scan to a previously identified axon, severing the axon shaft. Lesions were conducted in layer-1 of the cortex within 50 μm of the pial surface. Care was taken to lesion away from large vessels to avoid any confounding effects of the breakdown of the blood-brain-barrier. Complete axotomy was confirmed in each case by the eventual disappearance of the disconnected distal axon segment. The length of the disconnected axon was measured from the edge of the lesion site to the distal ending using Fiji software. Between 1 and 5 axons were selected for lesion in each brain, with lesions separated spatially and/or temporally, depending on the labelling pattern and density, maintained clarity of the imaging window and the number of anaesthetic/imaging sessions required.

### Repeated imaging of axon degeneration in vivo

Since it was not possible to predict when the axon fragmentation process would start, cortical axons were imaged before the lesion and at variable intervals after the lesion, until at least 50% of the disconnected axon had disappeared. On average, 89.6 ± 13.9% of the axon length disappeared over 5.5 time points per axon (range 3–21 imaging sessions, *n* = 43 in vivo cortical axons). To ensure the welfare of the animals over the prolonged imaging sessions, which lasted up to 6 days, while still ensuring accurate measurements of the fragmentation kinetics, imaging intervals were kept to less than 5 h between sessions (i.e. the maximum interval between any two consecutive sessions was 5 h). As a result, the average time interval recorded after fragmentation onset was 103 min. Therefore, the maximum fragmentation speed we could measure, on average, for a segment undergoing roWD is ~ 300 μm/103 min = ~ 3 μm/min, and for a segment undergoing WD, ~ 1000/103 = ~ 10 μm/min. This equates to a situation where the axonal segment is entirely removed between two consecutive time points. In a subset of cases, reducing the imaging interval after fragmentation onset, to an average of 13 min between images (with a minimum of 4 images collected during the fragmentation phase for any axon; average 9 images, range 4–16), however, did not significantly change the measured fragmentation rates (for < 600 μm length axons: 0.6 ± 0.1 μm/min; *n* = 8; *p* > 0.05 compared to segments < 600 μm imaged on average every 103 min) and onset (160 ± 24 min; *n* = 8 segments imaged with less than 2 h intervals; *p* > 0.05 compared to segments < 600 μm imaged with less than 4 h intervals), confirming our methodology. A subset of axons (*n* = 16 imaged with a membrane marker and 10 with an axoplasm one) were imaged more frequently for the first 60 min to probe for AAD. These experiments, which consisted of higher number of imaging time points acquired, also rule out any contribution of photo-bleaching of either membrane-bound or cytosolic GFP to the interpretation of degeneration kinetics.

After the final imaging session, mice were administered a lethal dose of ketamine/xylazine and transcardially perfused with ice-cold saline followed by 4% paraformaldehyde. Brains were removed and post-fixed overnight in paraformaldehyde at 4 °C before being transferred to 0.01 M PBS and stored in the fridge.

### Laminectomy and spinal cord imaging

Dorsal laminectomy was performed at lumbar spinal levels 1–3 prior to two-photon imaging in a non-recovery surgical procedure. Mice (Thy1-L15 *n* = 5, Thy1-GFPM *n* = 5) were anaesthetized with intraperitoneal injection of ketamine-xylazine mixture (0.083 mg/g ketamine, 0.0078 mg/g xylazine). After shaving the back, a longitudinal incision was made to retract the skin. The peri-vertebral muscles were deflected to expose the vertebral column and the laminae were removed using curved blade scissors. The mouse was then moved carefully to the microscope stage. The trunk was raised slightly and the vertebral column was clamped between two custom made bars for stabilisation during 2-photon imaging. The area surrounding the exposed spinal cord was circled by a layer of veterinary *n*-butyl cyanoacrylate adhesive to facilitate the formation of a saline meniscus between the objective and the exposed spinal cord. Labelled ascending axons in the dorsal white matter tracts were targeted for micro-lesion. The anaesthesia level was maintained by inhalation of an oxygen and isoflurane mixture (1.5–2.5%) throughout the imaging sessions. Mice were administered a lethal dose of ketamine/xylazine at the conclusion of the imaging session and transcardially perfused with ice-cold saline followed by 4% paraformaldehyde.

### Organotypic cortical slice culture preparation

Organotypic slices were prepared from the brains of neonatal transgenic mice (Thy1-L15, described above) based on [54]. Pups (*n* = 29) were killed at postnatal day (P) 3–4 by decapitation. Brains were carefully removed and immediately submerged in ice-cold culture medium (MEM Hanks supplemented with 20% heat-inactivated horse serum, Penicillin/Streptomycin (1%), Insulin (1 μg/ml), CaCL_2_ (1 mM), MgSO_4_ (2 mM), ascorbic acid (1.2%)). The cortical hemispheres were dissected away from the diencephalon and cortical slices (400 μm) were obtained with a McIlwain Tissue Chopper (Laboratory Engineering Co. Ltd., UK). Slices were carefully transferred, using a fire-polished, wide bore glass Pasteur pipette, to 35-mm petri dishes containing fresh, ice-cold culture medium and incubated on ice for 40 min. Using a dissection microscope, slices containing the somatosensory cortex were carefully transferred to Millicell culture plate inserts (Millipore) and incubated for up to 2 months with 1-ml culture medium at 35 °C in a humidified atmosphere with 5% CO2. The culture medium was changed twice weekly throughout the culture period.

### Imaging of axon degeneration in organotypic slice cultures

Organotypic slice cultures were imaged using two-photon microscopy beginning at 21–28 days in vitro (DIV). Culture inserts containing slices were transferred to sterile 35-mm petri dishes containing 1 ml pre-warmed Tyrodes salt solution (Sigma), and a further 1 ml was added on top of the slice. Imaging sessions of up to 1.5 h, using low power (< 20 mW at the back focal plane), were possible without noticeable deleterious effects in the slice. Typically 1–4 axons were chosen per slice (maximum length, 5 mm). Laser-mediated micro-lesions were performed as described for in vivo imaging protocols. To investigate the effects of NAD^+^ on the dynamics of axonal degeneration, slices were incubated for 24 h pre- or postlesion, with medium containing 1 mM NAD^+^ or with medium alone. Following imaging and/or lesioning, the salt solution was carefully removed from above and below the membrane and the inserts were returned to the incubator with fresh, pre-warmed culture medium (with or without NAD^+^, as appropriate).

### Experimental design and statistical analysis

Our experimental design involves longitudinal imaging to analyse cellular dynamics before and after a laser-mediated microlesion. The experimental design and statistical analysis of the data was discussed with in-house biostatisticians prior to commencement of analysis. Sample sizes have been set by power calculations in Pass software, using a significance threshold of 5% and a power of 80%. Previous experience of published experimental systems including ours indicates that data points are not normally distributed (normality tests were used) and tests compared two conditions within the same animals (before and after the lesion). Hence statistical significance was calculated by Mann-Whitney *U* test.

### Data analysis

Maximum projections of individual image stacks from in vivo and in vitro two-photon images of axons from all mice were processed with Fiji (file conversions, length quantifications) and Adobe Photoshop (section alignment, image rotation, brightness and contrast adjustment). Bouton density was determined by annotating *Terminaux* Boutons (TBs) in Matlab using custom made software [45, 84]. TBs (defined as protrusions between 1 and 5 μm) were scored according to stringent criteria based on [43] and [45]. In previous work, we performed correlated 2-photon : electron microscopy to confirm the presence of complete synaptic connections corresponding to imaged boutons along the same type of cortical axons as in the present study [38, 44, 45].

The resolution of our multiphoton microscope (0.25 × 0.25 × 2.5 in X-Y-Z) is sufficient to resolve gaps along the axons of few microns. Axons were excluded from analysis in cases where the visibility was compromised (e.g. by low window clarity, high axon labelling density or weak GFP signal).

The length of the distal portions from the edge of the lesion mask to the terminal ending was measured for all axons. ‘Ending’ and ‘close to the lesion’ were used to describe each end of the disconnected axon where ending refers to the terminal ending of the axon. Axons were grouped as either unbranched or branched, where the total length was less than or more than 600 μm.

Figures were prepared using Microsoft Office Suite, Adobe Illustrator and BioRender.

Statistical analyses were performed in Microsoft Excel, R or GraphPad Prism. Unless otherwise stated, all measurements are given as the mean ± standard error of the mean (SEM). Student’s *t* tests or Mann Whitney *U* tests were performed, unless otherwise stated. Results were considered significant when *p* < 0.05.

## Supplementary information

**Additional file 1. **Canty et al. Additional file 1.pdf. Examples of fragmenting cortical axons. (**a**, **b**) Representative images and tracings of two short axon endings undergoing rapid onset WD (roWD) with time post-lesion and percentage of axon lost indicated. (**c**) Representative images and tracings of a branched, longer axon undergoing Wallerian degeneration (WD) with time post-lesion and percentage of axon lost indicated. Dotted boxes refer to the axon segments shown below. Scale bar is 20 μm in a, 25 μm in b and 32 μm in c.

**Additional file 2.** Canty et al. Additional file 2.pdf. Probing the onset mechanism of lesioned axons. Schematic depiction of ‘protective factor’ and ‘degeneration trigger factor’ regulated axon degeneration paradigms following a single or double lesion. Lightning bolt represents lesion site. Purple arrows indicate movement of protective factor out of the lesioned axon via one (single lesion) or two routes (double lesion). Red arrows indicate movement of a degeneration trigger into the lesioned axon via one (single lesion) or two routes (double lesion).

** Additional file 4.** AAD involves axoplasmic but not axolemmal fragmentation in spinal cord sensory axons after axotomy. (**a**) Axoplasmic fragmentation, AAD, occurs after laser-mediated lesion of ascending sensory neurons expressing a cytosolic form of GFP (white arrowhead at + 65 min, *n* = 9 axons, 5 mice). (**b**) Axons remain intact for longer in ascending Thy1-L15 sensory axons expressing a membrane-bound form of GFP (n = 9 axons, 5 mice). Note beading of axons, but no fragments for up to ~ 2 h postlesion. (**c**) The mean onset of sensory axon fragmentation is significantly shorter for GFP-M (cytosolic GFP) compared to L15 (membrane-targeted GFP) axons. (**d**) Mean distance of severed proximal and distal axon tips to the lesion site in GFP-M (cytosolic GFP, n = 9 axons, blue lines) and L15 (membrane-targeted GFP, n = 9 axons, orange lines). Scale bar 50 μm for a and b. *** *p* < 0.001.

**Additional file 5.** Axon degeneration modalities. Schematic depicts the characteristics of axon degeneration for acute axonal degeneration in the spinal cord (AAD, green), rapid onset Wallerian degeneration in the cortex (roWD, blue) and Wallerian degeneration (WD) in the cortex (grey) and spinal cord (pink). From top to bottom, illustrated degeneration characteristics include the site of injury along the axon, the length of disconnected axon, the onset time at which fragmentation commences, and the fragmentation rate.

## Data Availability

All data generated or analysed during this study are included in this published article and supplementary information files.

## Abbreviations

AAD: Acute axonal degeneration
DIV: Days in vitro
GFP: Green Fluorescent Protein
NAD: Nicotinamide adenine dinucleotide
NMNAT: Nicotinamide mononucleotide adenylyl transferase
P: Postnatal day
PNS: Peripheral nervous system
roWD: Rapid-Onset form of Wallerian Degeneration
TB: *Terminaux* Bouton
WD: Wallerian degeneration
Wld^S^: Wallerian degeneration slow mouse
WT: Wild type
