# Supplementary figures and images for: In vivo imaging of injured cortical axons reveals a rapid onset form of Wallerian degeneration
Source: BMC Biol. 2020 Nov 18;18:170. doi: 10.1186/s12915-020-00869-2 (PMC7677840; doi:10.1186/s12915-020-00869-2)

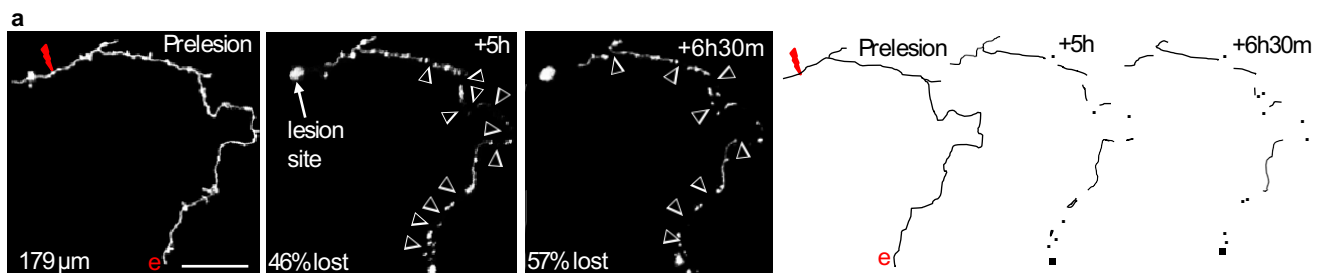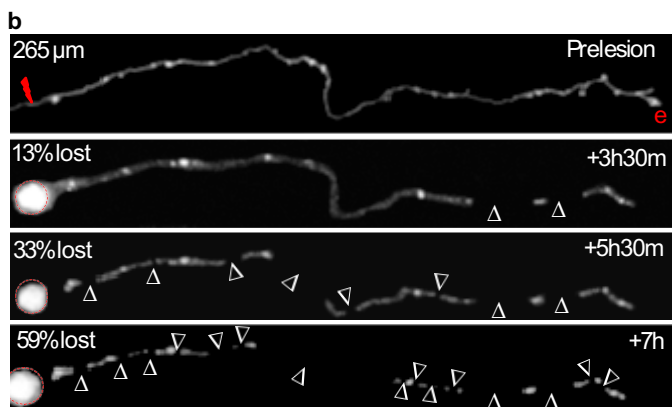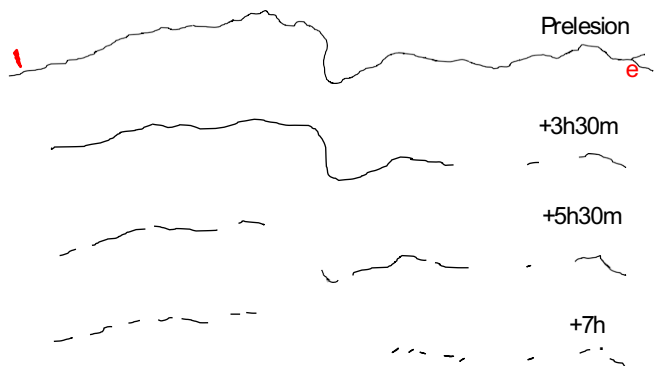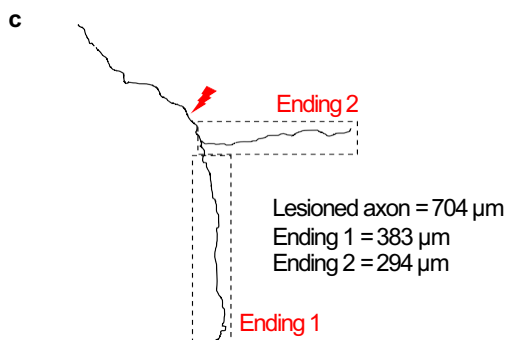

- lesion target
- lesion site
- axon ending
- close to lesion site
- fragmentation

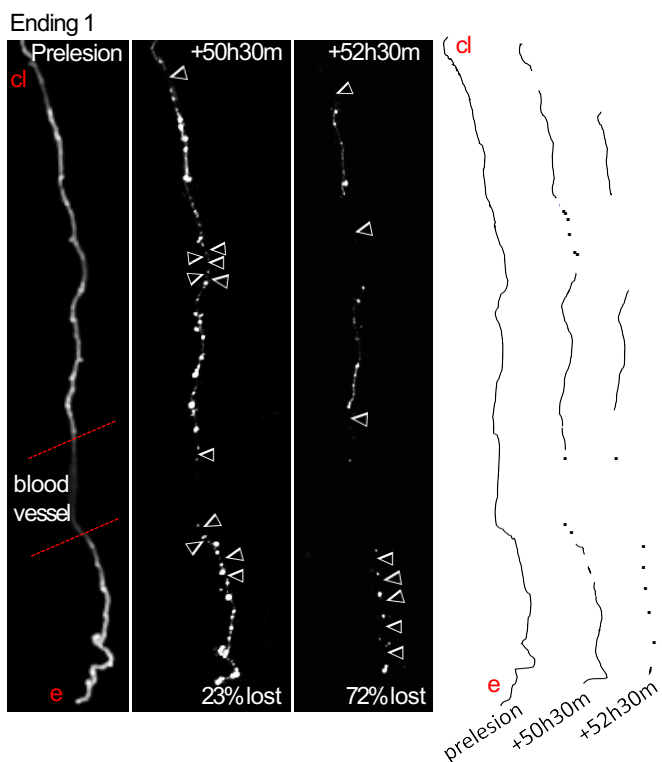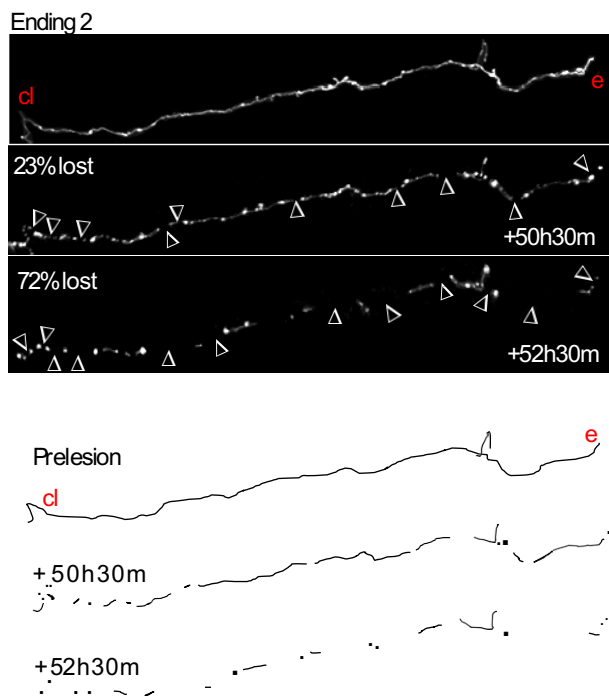

Supplement: Supplementary file 2 — Additional file 1. Canty et al. Additional file 1.pdf. Examples of fragmenting cortical axons. (a, b) Representative images and tracings of two short axon endings undergoing rapid onset WD (roWD) with time post-lesion and percentage of axon lost indicated. (c) Representative images and tracings of a branched, longer axon undergoing Wallerian degeneration (WD) with time post-lesion and percentage of axon lost indicated. Dotted boxes refer to the axon segments shown below. Scale bar is 20 μm in a, 25 μm in b and 32 μm in c. [file 12915_2020_869_MOESM1_ESM.pdf]

● Protective factor

● Degeneration trigger

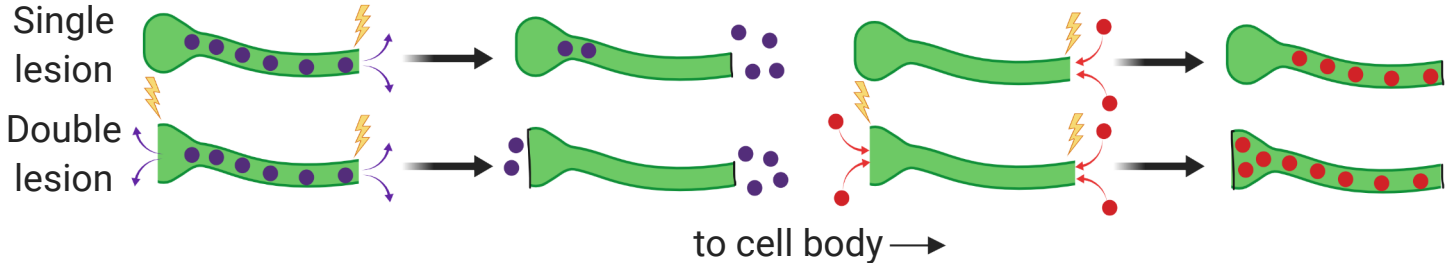

Supplement: Supplementary file 3 — Additional file 2. Canty et al. Additional file 2.pdf. Probing the onset mechanism of lesioned axons. Schematic depiction of ‘protective factor’ and ‘degeneration trigger factor’ regulated axon degeneration paradigms following a single or double lesion. Lightning bolt represents lesion site. Purple arrows indicate movement of protective factor out of the lesioned axon via one (single lesion) or two routes (double lesion). Red arrows indicate movement of a degeneration trigger into the lesioned axon via one (single lesion) or two routes (double lesion). [file 12915_2020_869_MOESM2_ESM.pdf]

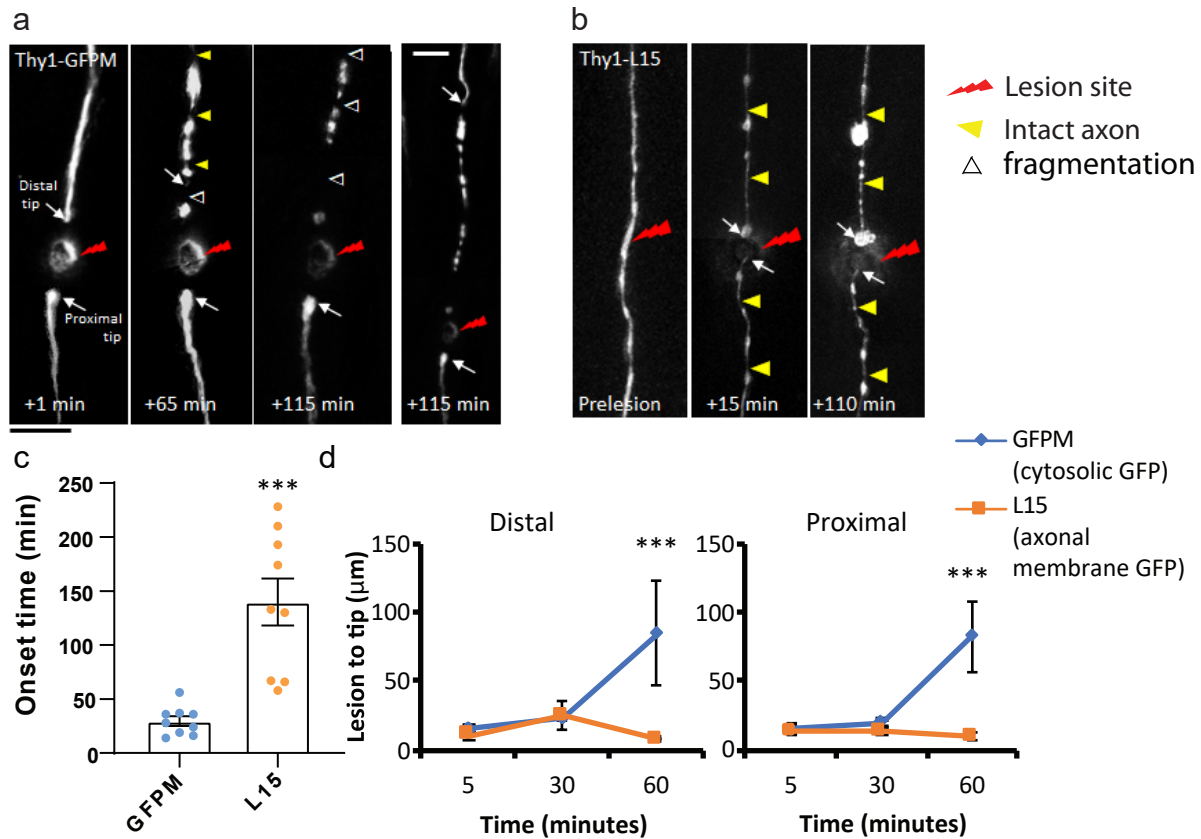

Supplement: Supplementary file 4 — Additional file 4. AAD involves axoplasmic but not axolemmal fragmentation in spinal cord sensory axons after axotomy. (a) Axoplasmic fragmentation, AAD, occurs after laser-mediated lesion of ascending sensory neurons expressing a cytosolic form of GFP (white arrowhead at + 65 min, n = 9 axons, 5 mice). (b) Axons remain intact for longer in ascending Thy1-L15 sensory axons expressing a membrane-bound form of GFP (n = 9 axons, 5 mice). Note beading of axons, but no fragments for up to ~ 2 h postlesion. (c) The mean onset of sensory axon fragmentation is significantly shorter for GFP-M (cytosolic GFP) compared to L15 (membrane-targeted GFP) axons. (d) Mean distance of severed proximal and distal axon tips to the lesion site in GFP-M (cytosolic GFP, n = 9 axons, blue lines) and L15 (membrane-targeted GFP, n = 9 axons, orange lines). Scale bar 50 μm for a and b. *** p < 0.001. [file 12915_2020_869_MOESM4_ESM.pdf]
