# Supplementary material for: In vivo imaging of injured cortical axons reveals a rapid onset form of Wallerian degeneration
Source: BMC Biol. 2020 Nov 18;18:170. doi: 10.1186/s12915-020-00869-2 (PMC7677840; doi:10.1186/s12915-020-00869-2)
Supplement: Supplementary file 5 — Additional file 5. Axon degeneration modalities. Schematic depicts the characteristics of axon degeneration for acute axonal degeneration in the spinal cord (AAD, green), rapid onset Wallerian degeneration in the cortex (roWD, blue) and Wallerian degeneration (WD) in the cortex (grey) and spinal cord (pink). From top to bottom, illustrated degeneration characteristics include the site of injury along the axon, the length of disconnected axon, the onset time at which fragmentation commences, and the fragmentation rate. [file 12915_2020_869_MOESM5_ESM.pdf]

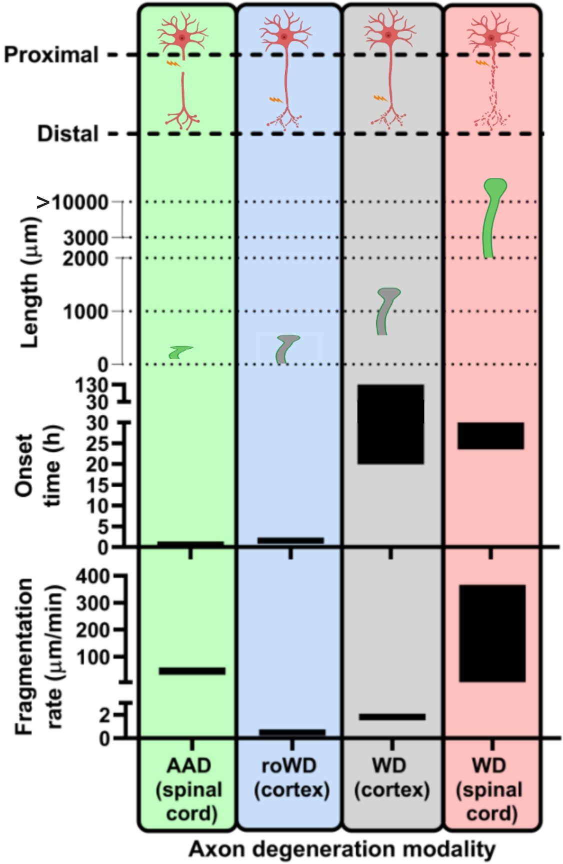

Proximal
Distal
>10000
3000
2000
1000
0
Length (µm)
130
30
30
25
20
15
10
5
0
Onset time (h)
400
300
200
100
2
0
Fragmentation rate (µm/min)
AAD (spinal cord)
roWD (cortex)
WD (cortex)
WD (spinal cord)
Axon degeneration modality
